# Structural Brain Changes Related to Disease Duration in Patients with Asthma

**DOI:** 10.1371/journal.pone.0023739

**Published:** 2011-08-19

**Authors:** Andreas von Leupoldt, Stefanie Brassen, Hans Jörg Baumann, Hans Klose, Christian Büchel

**Affiliations:** 1 Department of Psychology, University of Hamburg, Hamburg, Germany; 2 Department of Systems Neuroscience, University Medical Center Hamburg-Eppendorf, Hamburg, Germany; 3 Department of Pneumology, University Medical Center Hamburg-Eppendorf, Hamburg, Germany; Beijing Normal University, Beijing, China

## Abstract

Dyspnea is the impairing, cardinal symptom patients with asthma repeatedly experience over the course of the disease. However, its accurate perception is also crucial for timely initiation of treatment. Reduced perception of dyspnea is associated with negative treatment outcome, but the underlying brain mechanisms of perceived dyspnea in patients with asthma remain poorly understood. We examined whether increasing disease duration in fourteen patients with mild-to-moderate asthma is related to structural brain changes in the insular cortex and brainstem periaqueductal grey (PAG). In addition, the association between structural brain changes and perceived dyspnea were studied. By using magnetic resonance imaging in combination with voxel-based morphometry, gray matter volumes of the insular cortex and the PAG were analysed and correlated with asthma duration and perceived affective unpleasantness of resistive load induced dyspnea. Whereas no associations were observed for the insular cortex, longer duration of asthma was associated with increased gray matter volume in the PAG. Moreover, increased PAG gray matter volume was related to reduced ratings of dyspnea unpleasantness. Our results demonstrate that increasing disease duration is associated with increased gray matter volume in the brainstem PAG in patients with mild-to-moderate asthma. This structural brain change might contribute to the reduced perception of dyspnea in some patients with asthma and negatively impact the treatment outcome.

## Introduction

Dyspnea, which is a multidimensional respiratory sensation containing affective (unpleasantness) and sensory (intensity) aspects [1]–[4], is the impairing and threatening cardinal symptom millions of patients with asthma repeatedly experience over the course of the disease [5], [6]. However, accurate perception of dyspnea is also important for successful self-management and clinical treatment of asthma because it motivates patients to initiate appropriate health behavior such as seeking timely medical and self-treatment in adequate doses [4], [7]. It is well documented that the perception of dyspnea shows considerable variations between patients and is often not correlated with simultaneous lung function measurements [8], [9]. Importantly, reduced perception of initial bronchoconstriction and dyspnea in patients with asthma can lead to increased morbidity due to delayed or inadequate medication use, delayed visits to the physician or emergency department and might even result in near-fatal and fatal attacks which is often not related to patients' baseline lung function [10]–[14].

The blunted perception of dyspnea in some patients with asthma is still poorly understood and might involve psychological factors [15], [16], habituation processes [17]–[19], or reduced chemosensitivity [13] and seems to be linked to disease duration [15]. Few previous studies using electroencephalography (EEG) suggested reduced neural processing of dyspneic respiratory signals to be another potential cause of blunted dyspnea perception [18], [20]–[23]. Unfortunately, the current knowledge about brain processes underlying the perception of dyspnea is still considerably limited [24], [25]; particularly neuroimaging studies in patients with asthma are needed.

By using functional magnetic resonance imaging (fMRI) we previously demonstrated that patients with mild-to-moderate asthma reported reduced affective unpleasantness of resistive load induced dyspnea when compared to healthy controls [26]. This was mirrored by reduced insular cortex activations which were correlated with patients' asthma duration, but increased asthma-specific activations in the periaqueductal grey (PAG) during increasing dyspnea levels. The insular cortex is an important multisensory integration area and involved in the processing of various unpleasant bodily signals but also of emotions [27]–[29], whereas the PAG plays an important role in the up- and down-regulation of pain sensations but also in fear and defensive behaviour [30]–[32]. We interpreted these previous results as neural habituation to repeated dyspnea experiences over the course of disease that reduces the perceived affective unpleasantness of dyspnea in asthma [26]. However, whereas these findings demonstrated asthma-specific differences in functional responses of insular cortex and PAG, they did not allow conclusions about possible structural alterations in these brain areas which might have also developed over the course of disease and, thus, could have impacted the perception of dyspnea. This assumption is supported by previous findings from research on pain demonstrating structural alterations in pain processing brain areas in chronic pain patients which were correlated with disease duration and pain reports [33]–[37]. Similar structural brain changes were hypothesized in patients with asthma, but have not been tested [38].

Therefore, by using magnetic resonance imaging (MRI) in combination with voxel-based morphometry (VBM) the present study examined, whether increasing asthma duration is related to structural changes in insular cortex and PAG. Based on asthma-specific functional brain responses observed in our previous study [26], we expected decreased gray matter volume in the insular cortex, but increased gray matter volume in the PAG with increasing disease duration in the same sample of patients with mild-to-moderate asthma. We further hypothesized the gray matter volume in these areas to be related to the perception of dyspnea unpleasantness.

## Methods

### Ethics Statement

The study was approved by the medical ethics committee Hamburg and conducted according to the principles expressed in the Declaration of Helsinki. Written informed consent was obtained from all participants.

### Participants

We examined fourteen patients with mild-to-moderate asthma, based on criteria of the Global Initiative for Asthma [5] and fourteen healthy controls being matched for age and gender. All subjects participated in our previous study on functional brain responses to induced dyspnea [26], were free of major respiratory symptoms in the preceding 4 weeks and had to refrain from taking their asthma medication (except inhaled corticosteroids) for 16 hours prior to the study. Before testing, all participants were screened by questionnaires and a diagnostic interview to exclude significant psychological and medical conditions.

### Voxel-based morphometry

#### Image acquisition

High-resolution MR scanning was performed on a 3 Tesla Magnetom-TRIO MR scanner (Siemens Medical Solutions, Erlangen, Germany) with a standard head coil. A T1-weighted structural MRI was acquired for each participant by using a 3D-FLASH sequence (TR 15 ms, TE 4.9 ms, flip angle 15°, FOV 256×256, 192 slices, voxel size 1×1×1 mm). Due to the short echo time (4.9 ms), this sequence minimizes susceptibility artifacts which can be problematic in functional brain stem images using T2*-weighted sequences with longer echo times.

#### Image processing and statistical analysis

Data preprocessing and statistical analyses were performed with SPM8 (www.fil.ion.ucl.ac.uk/spm) using the voxel-based morphometry toolbox (VBM8; http://dbm.neuro.uni-jena.de/) that is based on high-resolution structural 3D MR images and allows for applying voxel-wise statistics in order to detect regional differences in gray matter volumes [39], [40]. All T1-weighted images were corrected for bias-field inhomogeneities, spatially normalized and segmented into gray matter, white matter, and cerebrospinal fluid within the same generative model [41], and spatially smoothed with a Gaussian kernel of 6 mm full-width-at-half-maximum. The segmentation procedure was further extended by accounting for partial volume effects [42], by applying adaptive maximum a posteriori estimations [43], and by applying hidden Markov random field model as described by Gaser [44].

To analyze the relationship between duration of asthma and gray matter changes in insular cortex and PAG, a single regression analysis based on a general linear model (GLM) as implemented in SPM8 was used for the patient group which included the individual disease duration (in years) as regressor. Based on our previous findings in functional brain responses [26], we examined first whether disease duration was correlated with decreased gray matter volume in the insular cortex. Second, we tested whether disease duration was correlated with increased gray matter volume in the PAG. Third, by using Spearman's rank correlations we examined whether gray matter volumes in areas with significant relation to disease duration were correlated with the ratings of perceived unpleasantness of resistive load induced dyspnea from our previous functional MRI study. These ratings were obtained immediately after functional scanning on a verbal descriptor list consisting of fourteen affective adjectives, which were rated on a 4 point-Likert scale and condensed to a summary score [26]. Finally, we examined the possibility that structural changes in the patient group were merely results of normal aging-related changes in brain structure [40] by running a control analysis in the healthy control group with age as regressor. Specifically, we tested whether age was correlated with gray matter changes in those areas that showed a significant relation to disease duration in the patients with asthma. The threshold for statistical significance was set to p<0.05, corrected for multiple comparisons. Regions of interest (ROIs) were spheres of 6 mm radius around the following center coordinates: ±39, 3, −2 (insular cortex) and 0, −21, −6 (PAG). Coordinates were chosen from our previous functional imaging study [26].

## Results

According to GINA guidelines [5], studied patients had mild-intermittent (N = 4), mild-persistent (N = 6), and moderate-persistent (N = 4) asthma. The mean duration of asthma was 16.4 (SD: 8.8) years. No differences in baseline characteristics were found between patients and controls, except lower FEV_1_ in patients (Table 1).

**Table 1:** Mean (SD) baseline characteristics of participants.

	Patients with asthma	Healthy controls
Age (yr)	27.0 (4.2)	26.6 (6.2)
Sex (female/male), No.	7/7	7/7
Weight (kg)	67.1 (13.4)	66.6 (11.5)
Height (cm)	171.7 (10.1)	174.8 (9.1)
Body Mass Index (kg/m^2^)	22.6 (2.6)	21.7 (2.2)
Disease duration (yr)	16.4 (8.8)	-
FEV_1_ (L)	3.83 (.74)	4.35 (1.05)
FEV_1_ (% predicted)	100.7 (8.8)	110.9 (15.7)†
FVC (L)	4.79 (1.11)	5.27 (1.18)
FVC (L) (% predicted)	106.9 (10.8)	114.4 (10.1)

FEV_1_ = forced expiratory volume in 1s, FVC = forced vital capacity.

† p<0.05 (t-tests) for the comparison between patients with asthma and healthy controls.

**Figure 1:**
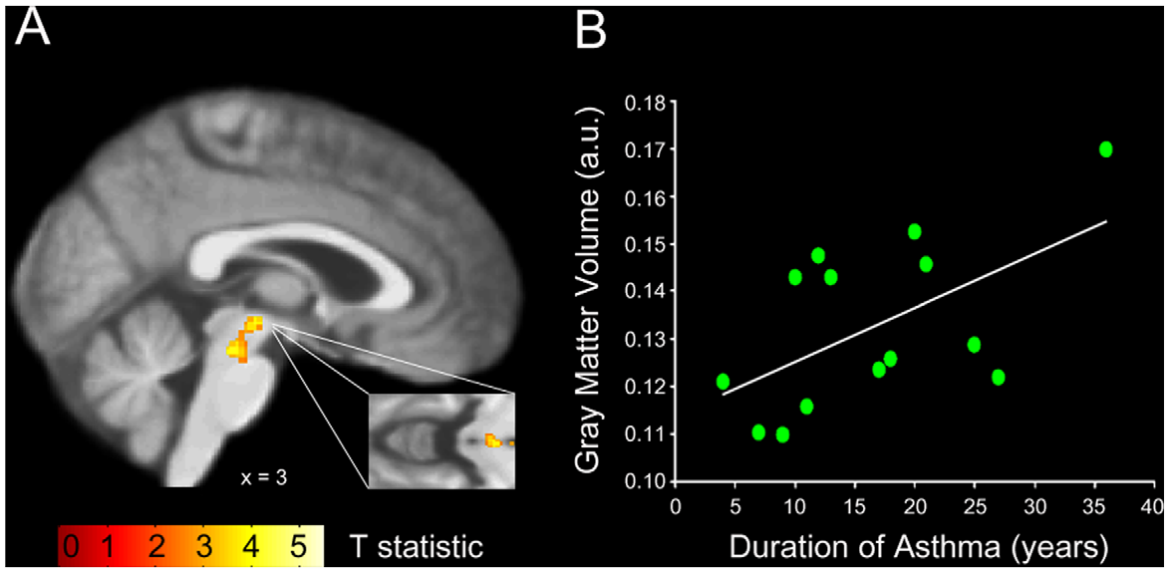
PAG gray matter volume in relation to the duration of asthma in the patient group. A) Statistical parametric map demonstrating a significant increase of gray matter volume in the brainstem PAG with increasing duration of asthma (shown at p<0.005, uncorrected). Gray matter changes are superimposed onto the asthma groups' mean structural T1 weighted MRI image. B) Mean PAG gray matter volumes of each patient in relation to the individual duration of asthma.

**Figure 2:**
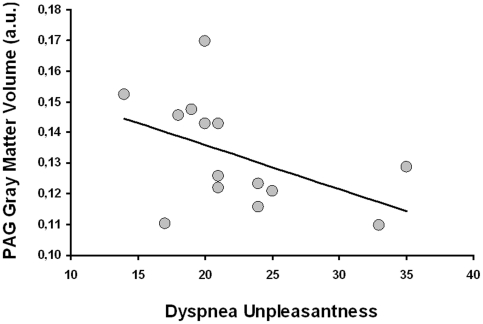
PAG gray matter volume in relation to ratings of dyspnea unpleasantness in patients with asthma. A significant negative correlation (r = −0.51, p<0.05) demonstrated that increased mean PAG gray matter volume in patients with asthma was associated with reduced ratings of perceived affective unpleasantness of resistive load induced dyspnea.

**Figure 3:**
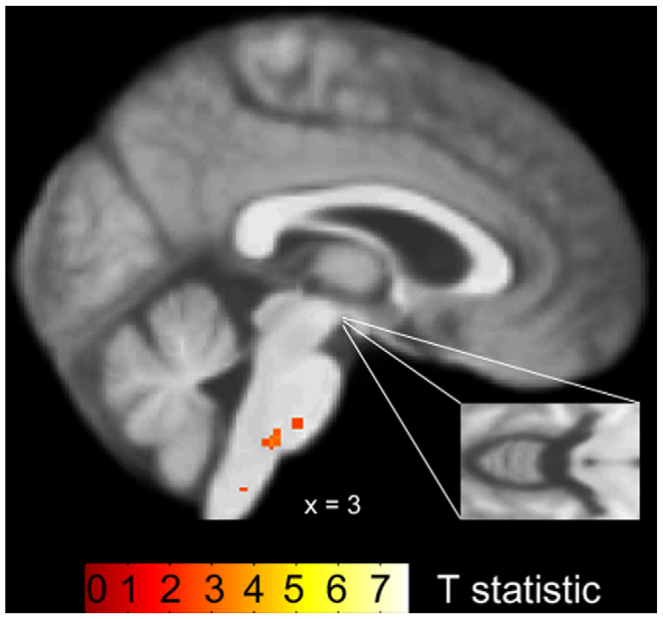
PAG gray matter volume in relation to age in the control group. Statistical parametric map demonstrating no increase of gray matter volume in the brainstem PAG with increasing age (shown at p<0.005, uncorrected). Gray matter changes are superimposed onto the control groups' mean structural T1 weighted MRI image.

In the patients with asthma, no significant associations between gray matter volume of the insular cortex and disease duration were observed. However, for the bilateral PAG we found a significant increase in gray matter volume with increasing disease duration (MNI coordinates of peak voxels: 3, −21, −9; Z = 3.22; p<0.05 and −3, −22, −9; Z = 3.15; p<0.05; Figure 1). As demonstrated in Figure 2, a significant negative correlation (r = −0.51, p<0.05) in the patient group showed that increased gray matter volume in the PAG was associated with reduced reports of affective unpleasantness of resistive load induced dyspnea. In the healthy control group, no associations were observed between gray matter volume in the PAG and age or dyspnea unpleasantness ratings, respectively (Figure 3).

## Discussion

Using voxel-based morphometry, the present study shows that longer duration of asthma was associated with increased gray matter volume in the brainstem PAG in patients with mild-to-moderate asthma. This finding seems unrelated to changes in brain structure caused by normal aging [40] because no age-related PAG gray matter volume changes were observed in a well matched healthy control group. Moreover, increased gray matter volume in the PAG in patients with asthma was related to reduced ratings for the perceived affective unpleasantness of resistive load induced dyspnea. Thus, the present findings demonstrate that increasing disease duration is associated with structural brain changes in patients with asthma which, in turn, are related to reduced perception of the affective dyspnea unpleasantness. These alterations in a brain structure with a well documented role in the antinociception of pain [30], [31] and in fear and defence behaviour [32], are therefore likely to contribute to the blunted perception of dyspnea at least in subgroups of patients with asthma.

The present results are in line with our previous study in the same sample of patients that demonstrated asthma-specific reductions in perceived dyspnea unpleasantness to be mirrored by increased functional responses of the PAG during increasing levels of dyspnea [26]. This was paralleled by asthma-specific reductions in insular cortex activations during increasing dyspnea which were also correlated with disease duration and were moderated by increased PAG activity. However, in the present study we did not observe significant structural changes in the insular cortex that were related to the duration of asthma. Therefore, it might be speculated that decreased insular cortex activations are the result of structural changes in the PAG with increasing disease duration which lead to increased antinociceptive PAG activity. Increased PAG activity, in turn, might lead to increased inhibition of throughput of dyspneic sensory afferences to the insular cortex and result in reduced insular activations without structural changes of this area. However, we cannot exclude the possibility that the limited sample size reduced the test power to detect more subtle gray matter changes also in the insular cortex.

Our present findings are converging with earlier studies using EEG which similarly showed asthma-specific functional alterations in the brain processing of respiratory sensations. Specifically, these studies demonstrated attenuated or even absent respiratory-related evoked potentials to brief inspiratory occlusions in paediatric and adult asthma patients [20], [21], [23], which were partly related to reduced perception of the respiratory stimuli [20]. The present results suggest that structural brain changes might contribute to these alterations in functional brain responses. Given earlier findings that demonstrated blunted perception of dyspnea to be associated with inadequate or delayed treatment including near-fatal and fatal attacks [10]–[14], the present structural, but also the previously reported functional brain alterations might constitute an important risk factor in asthma. Particularly, the present association of increased PAG gray matter volume with decreased dyspnea unpleasantness ratings might be critical, because the affective unpleasantness of perceived dyspnea has been suggested as being specifically relevant for motivating patients with asthma to timely take effective medication or to seek professional help [4], [7].

However, although we cannot specify the exact mechanisms underlying the observed structural brain changes in the PAG in our patient sample, the most plausible mechanism seems to be a neuroplastic response of this area to repeated dyspnea experiences over the course of disease. This converges with our present observation that asthma duration was correlated with increased PAG gray matter volume, but also with reduced insular cortex responses during increasing dyspnea in our previous functional MRI study [26]. Similarly, reduced functional neural responses to short inspiratory occlusions were recently demonstrated in healthy volunteers when occlusions were repeatedly presented over the course of an EEG experiment [18]. Moreover, animal models showed that extended repeated exposures to stimuli mimicking allergic or mechanical asthma symptoms can result in functional plasticity in respiration related brain areas [45]–[47]. For example, Davenport and colleagues demonstrated state changes in neural activity in several brain areas including the PAG as well as changes in gene expression profiles following 10 days of chronic intermittent tracheal occlusions in rats [46], [47].

Further support comes from previous studies on chronic pain demonstrating gray matter changes in patients with various pain syndromes [33]–[37]. For example, Rocca and colleagues [35] reported similar increases in PAG gray matter volume in patients with repeated experiences of migraine attacks compared to controls. Most of these studies demonstrated the structural brain changes in chronic pain patients to be related to longer disease duration and, thus, longer experience with painful nociceptive input [33]–[35]. Moreover, gray matter changes in pain patients were shown to be correlated with reports of perceived pain [33], [37] which converges with the present observation of a correlation between PAG gray matter volume and perceived dyspnea unpleasantness. Interestingly, a previous study in chronic pain patients demonstrated that interruption of painful nociceptive input due to successful treatment not only leads to reduced pain reports, but also to a reversal of gray matter abnormalities [36]. In this regard, it will be interesting to examine in controlled longitudinal studies whether successful compared to unsuccessful or no treatment of dyspnea in patients with asthma or other chronic respiratory diseases might lead to a similar reversal in gray matter alterations.

However, morphometric techniques such as VBM do not allow conclusions about the specific intracranial processes that led to the neuroplastic responses observed in the present study, but also in studies on pain or other sensory experiences [48]. In general, gray matter increases could be due to various mechanisms including axonal remodeling, growth of new dendritic spines, synapse turnover, an increase in cell size, neural or glial cell genesis, spine density or even changes in blood flow or interstitial fluid, which all have been suggested to contribute to experience-dependent cortical plasticity [49]–[53]. Therefore, future studies with different methodology will be necessary to examine which of these potential mechanisms contribute to structural brain changes related to disease duration in patients with asthma.

When interpreting the present results some limitations should be kept in mind. Based on our previous observations of asthma-specific functional brain responses in the PAG and insular cortex in a rather small sample of patients, we limited the regions of interest to these two areas in the present study. This might have prevented the detection of further or more subtle gray matter changes in other brain areas involved in the processing of dyspnea. Future studies with larger samples are therefore required. Because we only examined patients with mild-to-moderate asthma, these studies might profit from including patients with more severe forms of the disease in order to examine structural brain alterations in relation to asthma severity. In this regard, it would be highly informative to include additional outcome measures that closely mirror the course of the disease such as frequency of exacerbations, hospitalisations or emergency treatments which were not available in the present study. Furthermore, effects of long-term use of asthma medication on the perception of dyspnea have been suggested [54], [55] and we cannot rule out respective effects on the underlying brain structures including the PAG. Therefore, future controlled studies should try to examine the impact of asthma medication on brain structure. Finally, the present study can not draw definite conclusions whether structural brain changes precede altered functional brain responses or vice versa, which clearly necessitates future studies using longitudinal designs.

In summary, the present findings demonstrate that increasing disease duration is associated with increased gray matter volume in the brainstem PAG in patients with mild-to-moderate asthma which is related to reduced perception of the affective unpleasantness of dyspnea. Future studies are required to examine whether these structural brain alterations are related to blunted perception of dyspnea, negative course of disease, asthma severity and/or asthma medication.
